# HL-Mamba: A High–Low Frequency Interaction Mamba Network for Hyperspectral Image Classification

**DOI:** 10.3390/s26051556

**Published:** 2026-03-02

**Authors:** Yehong Teng, Shu Gan, Xiping Yuan

**Affiliations:** 1 School of Land and Resources Engineering, Kunming University of Science and Technology, Kunming 650093, Chinayxp_kust@163.com (X.Y.); 2 Application Engineering Research Center of Spatial Information Surveying and Mapping Technology in Plateau and Mountainous Areas Set by Universities in Yunnan Province, Kunming 650093, China

**Keywords:** hyperspectral image classification, high–low frequency decomposition, cross-frequency interaction, frequency alignment loss, deep learning

## Abstract

Deep-learning-based methods have achieved remarkable success in hyperspectral image (HSI) classification tasks due to their promising ability. However, the high dimensionality and spectral–spatial correlations of HSIs usually lead to information redundancy and feature entanglement, limiting the classification performance. To address these issues, we propose a novel high–low frequency interaction Mamba network, called HL-Mamba, which achieves effective decoupling and interaction between global structures and edge details of HSIs in the frequency domain, thereby improving spectral–spatial representation for HSI classification. Specifically, a high–low frequency decomposition Mamba module is designed to decompose the HSI into low-frequency structural and high-frequency edge detail components, which allows the model to learn global structures and fine-grained details, enhancing classification performance. By employing two parallel Mamba branches to model long-range dependencies across different frequency components, the network achieves efficient global modeling while mitigating information redundancy. Furthermore, a cross-frequency interaction module is designed to establish complementary information flow between high- and low-frequency features through a dynamic attention mechanism. In this way, low-frequency structural features guide the aggregation of high-frequency details, whereas high-frequency textures refine global structural representations, yielding more discriminative spectral–spatial features for HSI classification. In addition, a frequency alignment loss is designed to enhance the consistency and complementarity between high- and low-frequency features, further improving classification performance. Extensive experiments on four public benchmark datasets (i.e., Indian Pines, Pavia University, WHU-Hi-HanChuan, and Houston datasets) demonstrate that the proposed HL-Mamba significantly outperforms eight comparison methods, achieving an overall accuracy of 94.07%, 93.82%, 95.28%, and 87.32%, respectively. Ablation studies further verify the effectiveness of core component within the network.

## 1. Introduction

Hyperspectral imaging technology has emerged as one of the most significant research topics in remote sensing, as it provides rich spectral and spatial information through hundreds of contiguous spectral bands [1,2]. The remarkable potential of hyperspectral images (HSIs) plays a pivotal role in diverse applications, such as environmental monitoring [3], agricultural management [4], mineral exploration [5], and urban planning [6]. HSI classification serves as the most critical step in many of these applications, aiming to assign a distinct land cover category to each pixel [7].

Over the past decade, numerous methods have been proposed to enhance the performance of HSI classification [8]. Early methods mainly relied on traditional machine learning techniques such as support vector machines [9], k-nearest neighbors [10], and random forests [11], which primarily focused on spectral information. However, these methods often ignored spatial correlations, leading to spectral confusion and classification errors in complex scenes. To mitigate this limitation, spatial–spectral feature extraction methods are introduced, incorporating local spatial context via morphological profiles, Markov random fields [12], or principal component analysis (PCA)-based dimensionality reduction [13]. Nevertheless, these hand-crafted spatial–spectral feature extraction strategies still suffer from limited capability to adaptively capture the complex nonlinear relationships embedded in HSI data.

With the rapid advancements in deep learning, numerous deep-learning-based models have been proposed to improve HSI classification by jointly learning hierarchical spectral–spatial representations, which have shown significant superiority over traditional hand-crafted feature methods [14,15]. Among them, convolutional neural networks (CNNs) have gained significant attention due to their strong ability to capture local spatial–spectral dependencies of HSIs [16]. For instance, 2D CNNs and 3D CNNs are widely employed to learn hierarchical feature representations from pixel values and spectral reflectance sequences [17]. Yang et al. proposed an enhanced multiscale feature fusion network, which extracts multiscale features from parallel multipath structures across three stages to boost classification performance [18]. In addition to CNNs, recurrent neural networks and long short-term memory networks have been applied to HSI data to model sequential dependencies along the spectral dimension, fully leveraging the intrinsic correlation between contiguous spectral bands [19,20,21]. More recently, Transformer-based models, known for their powerful self-attention mechanisms, have also been adapted for HSI classification, achieving promising results by capturing long-range dependencies between spectral bands and spatial regions [22,23]. For example, Yang et al. proposed a center-to-surrounding interactive learning network via integrating Transformer architecture for more effective spectral–spatial feature learning [24]. However, the self-attention mechanism in standard Transformers incurs a quadratic computational complexity with the number of pixels, resulting in substantial computational redundancy and limiting its efficiency in processing HSI data.

To alleviate the above computational redundancy issue, subsequent studies have explored a series of efficient model designs, including CNN variants with depthwise separable convolutions [25], compact RNN architectures with gated mechanisms, and improved Transformer models with sparse attention or linear attention [26]. These methods reduce computational overhead for HSI classification tasks, enabling efficient processing of high-dimensional HSI data [27]. Meanwhile, state space models (SSMs) represented by Mamba have also been introduced into HSI classification recently, due to their linear computational complexity and efficient long-range dependency modeling capability. For instance, Wang et al. proposed MambaHSI+, a framework that integrates bidirectional state-space modeling with spectral trajectory learning for HSI classification [28]. Liu et al. proposed a dual-classification-head self-training network that performs class-wise feature alignment across domains, achieving strong cross-domain classification performance [29]. Despite their success in efficiency and accuracy, these deep-learning-based methods still face nonnegligible limitations. They rely on complex network architectures and large-scale training data, and more importantly, they inherently entangle global structural features with fine-grained edge details in the feature learning process, leading to redundant and suboptimal feature representations, and making it difficult to simultaneously capture both global structural information and local fine-grained textures.

To address the feature entanglement between global structure and local details, frequency-based methods have recently emerged as a promising paradigm for HSI classification, by explicitly decomposing HSI data into different frequency components corresponding to structural and detail information, respectively [30]. Early attempts at frequency-based analysis for HSI classification mainly adopted Fourier transform and wavelet transform to decompose HSI into multiresolution frequency components, enabling multiscale feature extraction [31]. Wavelet-based methods have shown unique advantages in handling the high dimensionality of HSIs, as they support the decomposition of spectral and spatial features at different scales [32]. However, these methods typically rely on hand-crafted frequency decomposition and feature extraction strategies, which may limit their generalization and performance when dealing with complex and diverse HSI datasets. To overcome this limitation, recent studies have combined deep learning techniques with frequency-domain representations, integrating frequency decomposition into end-to-end networks [33]. These frequency-based deep learning models take the decomposed frequency components as part of the input to CNNs, enabling the model to learn discriminative frequency-domain representations in an end-to-end manner [34], and have achieved significant improvements in classification accuracy [35]. For example, Yang et al. proposed the ITER method for weakly supervised HSI classification, which employs a high-frequency-aware self-attention in a high-enhanced transformer to refine detailed feature representation for pixel-level prediction [36]. However, a critical limitation of existing frequency-based methods is the lack of effective interaction between different frequency components, failing to fully exploit the complementary properties of high- and low-frequency features.

To address aforementioned core challenges of computational redundancy, feature entanglement between global structure and local details, and insufficient cross-frequency interaction in existing methods, we propose a high–low frequency interaction Mamba network (HL-Mamba) framework, tailored for HSI classification. This framework innovatively integrates frequency-domain feature decoupling with the efficient long-range modeling capability of Mamba to tackle the core pain points in HSI classification. First, a high–low frequency decomposition Mamba module is designed to decompose HSI into low-frequency structural and high-frequency detail components. The low-frequency branch efficiently captures long-range dependencies in global structures, while the high-frequency branch focuses on modeling fine-grained textures and edges. This frequency-wise separation manner enables the network to learn complementary features without mutual interference between global structural and edge details. Furthermore, a dynamic cross-frequency interaction module is proposed to establish bidirectional information flow. Thus, low-frequency structural features guide high-frequency detail aggregation, while high-frequency textures refine global structural representations, yielding more discriminative spectral–spatial features for HSI classification. To enhance feature consistency, and complementarity, a frequency alignment loss is designed, enhancing the classification performance. The main contributions of this article can be summarized as follows:

1. A high–low frequency interaction Mamba network is proposed, which innovatively integrates frequency-domain feature decoupling with the efficient long-range modeling capability of Mamba to tackle the core issues of feature entanglement and computational redundancy in HSI classification.
2. A dynamic cross-frequency interaction module is designed, where low-frequency structural features guide aggregation of discriminative high-frequency details and high-frequency textures refine global structural representations, yielding more discriminative spectral–spatial features for HSI classification.
3. A frequency alignment loss is designed to constrain the consistency and complementarity of distribution between the high- and low-frequency components, further enhancing the discriminability of classification.

## 2. Materials and Methods

### 2.1. Dataset Description

Four benchmark datasets are selected to ensure experimental diversity across different sensors. The detailed number of training and testing samples is summarized in Table 1.

1. Indian Pines dataset [37]: Captured over northwestern Indiana, USA, this dataset has a spatial resolution of 20 m and 220 spectral bands. The spatial size of image is 145 × 145 pixels, containing 16 land-cover classes dominated by agricultural crops (e.g., corn, soybean), forests, and low-density residential areas. The strong spectral similarity between corn, soybean, and grass species poses a significant challenge for HSI classification.
2. Pavia University dataset [38]: Acquired by the ROSIS sensor over Pavia, Italy, this dataset features a high spatial resolution of 1.3 m and 103 spectral bands. A spatial size of 610 × 340 pixels and 9 urban surface classes (e.g., asphalt, meadows, bare soil, self-blocking bricks), it is ideal for evaluating the fine-grained spatial discrimination capability of models due to its rich spatial details.
3. Houston dataset [39]: Released by the IEEE GRSS Data Fusion Contest 2013, this dataset includes 349 × 1905 pixels and 144 spectral bands, covering 15 complex urban and suburban classes (e.g., roads, parking lots, vegetation, buildings). High intra-class variability and mixed pixels caused by shadows and structural occlusions make it a challenging benchmark for urban HSI classification.
4. WHU-Hi-HanChuan dataset [40]: A large-scale dataset captured by the Wuhan University airborne hyperspectral sensor over Hanchuan, China. It covers 1100 × 5100 pixels with 270 spectral bands and a fine spatial resolution of 1 m. The 16 land-cover categories include agricultural crops (strawberry, soybean, sorghum), vegetation, water bodies, and built-up areas. The vast scene size and high spectral diversity enable a rigorous test of the model’s generalization to real-world scenarios.

### 2.2. Data Processing Methods

To ensure fair comparison with HSI classification methods, we adopt the widely accepted training–test split protocols consistent with mainstream works for the four datasets, randomly selecting a fixed proportion of labeled samples per land cover category as the training set and using remaining labeled samples as the independent test set, with the random seed fixed throughout the splitting process to guarantee result reproducibility and avoid data leakage. For data preprocessing, we first perform per-band min–max normalization on raw HSI data to map reflectance values to the range of [0, 1]. We then apply PCA with the transformation matrix fitted only on the training set. To construct standardized model inputs, we adopt a center-pixel neighborhood cropping strategy with a uniform spatial window size of K=7 for all datasets to balance local spatial context capture and computational efficiency, and use padding for edge pixels to supplement missing neighborhood information and ensure valid input patches for all pixels without introducing extra noise.

## 3. Proposed Method

In this section, the details of the HL-Mamba are presented. The core idea is to separate HSI into low-frequency and high-frequency components, enabling complementary feature learning via the Mamba network. A cross-frequency interaction module is also designed for feature interaction and learning.

### 3.1. Mamba: State Space Model for Sequence Modeling

Mamba, a novel state space mode proposed for sequence modeling, has recently shown remarkable superiority in processing long-range sequential data due to its linear computational complexity (O(N)) and efficient state propagation mechanism [28]. Different from traditional transformer-based architectures relying on self-attention, Mamba captures temporal and spatial dependencies by modeling the evolution of hidden states through a continuous-time SSM, which is particularly suitable for HSI classification tasks where spectral–spatial features exhibit sequential characteristics along spectral bands.

The core of Mamba is the discrete-time state space update, defined as:

(1) $$h_{t}=Ah_{t-1}+Bx_{t},\;y_{t}=Ch_{t}+Dx_{t}$$

where $h_{t}\in R^{d}$ denotes the hidden state at position *t*, $x_{t}\in R^{d}$ is the input feature, $y_{t}\in R^{d}$ represents the output, and $A\in R^{d\times d}$, $B\in R^{d\times d}$, $C\in R^{d\times d}$, $D\in R^{d}$ are learnable parameter matrices. To adapt to HSI’s multidirectional feature propagation, the diagonalized version of A (denoted as $\bar{A}$) is adopted to accelerate computation, leading to the simplified inference formula:

(2) $$h_{t}=\mathrm{diag}\left(\exp\left(\bar{A}\right)\right)h_{t-1}+Bx_{t}$$

where exp(·) denotes element-wise exponential operation and diag(·) converts a vector to a diagonal matrix. This design enables Mamba to efficiently model the long spectral sequences of HSI pixels while maintaining low computational cost, making it a promising backbone for HSI classification.

### 3.2. Overall Framework

**Figure 1:**
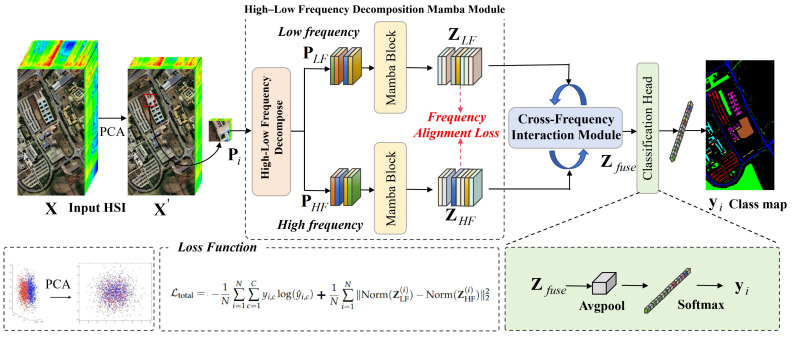
Architecture of the proposed HL-Mamba. The input HSI X is first processed by PCA to obtain $X^{\prime }$. Then, high–low frequency decomposition is performed to get $P_{LF}$ and $P_{HF}$. After Mamba modeling, $Z_{LF}$ and $Z_{HF}$ are generated and input into the cross-frequency interaction module to obtain $Z_{fuse}$. Finally, the classification result is output through the classification head. The loss function includes cross-entropy loss and frequency alignment loss, where λ is the balance weight.

HSI classification faces two core challenges, i.e., spectral–spatial feature entanglement and computational redundancy in long-range dependency modeling. Traditional HSI classification methods mainly rely on convolutions or self-attention mechanisms for feature learning [17,23]. However, convolutions are limited in capturing long-range dependences, while self-attention suffers from quadratic computational complexity. In addition, these methods treat the spectral–spatial features of HSIs as spatial domain, ignoring the inherent frequency properties of HSIs. Different frequency components preserve sharp edges and global structural features that are crucial for distinguishing similar classes. To address the above issues, an HL-Mamba is proposed (Figure 1). It integrates frequency-domain decomposition and long-range modeling of Mamba to explicitly process high- and low-frequency features. This reduces feature entanglement and computational redundancy, while boosting the discriminability of spectral–spatial representations for HSI classification.

Given an input HSI cube $X\in R^{H\times W\times B}$, where *H*, *W*, and *B* denote the spatial height, width, and number of spectral bands, respectively, we first apply principal component analysis (PCA) [41] to reduce spectral redundancy and preserve the most informative bands:

(3) $$X^{\prime }=\mathrm{PCA}\left(X\right),\;X^{\prime }\in R^{H\times W\times B^{\prime }}$$

where $B^{\prime }\ll B$ represents the reduced spectral dimension.

For pixel-wise HSI classification, 3D patches centered at each pixel are extracted as input samples:

(4) $$P_{i}\in R^{K\times K\times B^{\prime }}$$

where *K* denotes the spatial window size and $P_{i}$ is the local spatial–spectral cube of the *i*-th sample.

Each patch is processed by the proposed HL-Mamba, whose core components include a high–low frequency decomposition Mamba module, a cross-frequency interaction module, and a classification head. The overall forward process is defined as:

(5) $$y_{i}=f_{\mathrm{cls}}\left(f_{\mathrm{CFI}}\left(f_{\mathrm{LF}}\left(P_{i}\right),f_{\mathrm{HF}}\left(P_{i}\right)\right)\right)$$

where $f_{\mathrm{LF}}\left(\cdot \right)$ and $f_{\mathrm{HF}}\left(\cdot \right)$ denote the low- and high-frequency feature extraction branches, $f_{\mathrm{CFI}}\left(\cdot \right)$ represents the cross-frequency interaction module, and $f_{\mathrm{cls}}\left(\cdot \right)$ is the final classification head output $y_{i}\in R^{C}$ with *C* classes corresponding to the number of land-cover types.

This framework achieves frequency decomposition, long-range feature modeling, and adaptive interaction, ensuring that the model can simultaneously capture fine-grained edge features and global structural information, thus achieving high-precision HSI classification.

### 3.3. High–Low Frequency Decomposition Mamba Module

**Figure 2:**
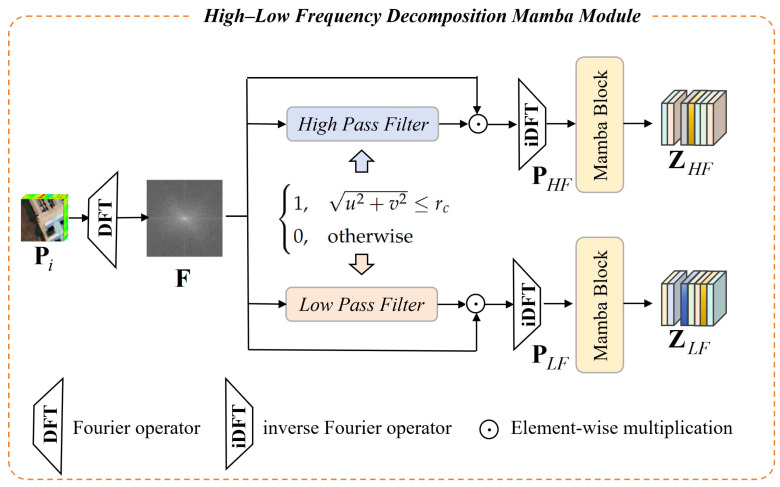
Architecture of the proposed high–low frequency decomposition Mamba module. An HSI patch $P_{i}$ is transformed to frequency domain by 2D DFT, split into low-frequency and high-frequency components via learnable masks with adaptive $r_{c}$, reconstructed to spatial domain by inverse DFT, and processed with two Mamba branches to output enhanced $Z_{\mathrm{LF}}$ and $Z_{\mathrm{HF}}$ for cross-frequency interaction.

HSI contains rich but redundant spectral–spatial information, and directly feeding deep networks leads to entangled feature learning. Moreover, the complex spatial–spectral correlation in HSI requires the model to have strong long-range dependency modeling capabilities, but traditional methods (convolutions or self-attention) are difficult to balance modeling effectiveness and computational redundancy. To address these problems, a high–low frequency decomposition Mamba module is designed (Figure 2), which first explicitly disentangles HSI to global structural representations and fine-grained texture representations, and then employs two parallel Mamba branches to model long-range dependencies under different frequency components.

Given a patch $P_{i}$, we perform a 2D discrete Fourier transform (DFT) along the spatial dimensions to convert the spatial domain information into the frequency domain:

(6) $$F=F\left(P_{i}\right),\;F\in C^{K\times K\times B^{\prime }}$$

where F(·) is the DFT operator. In the frequency domain, each spatial frequency component corresponds to different levels of spatial variation and structural smoothness. Low-frequency components are distributed in the central region, corresponding to smooth global structures and semantic information, while high-frequency components are distributed in the peripheral region, corresponding to sharp edges and local texture details.

To accurately separate high- and low-frequency components, we define two complementary frequency masks based on the distance from the frequency domain center:

(7) $$M_{\mathrm{LF}}\left(u,v\right)=\left\{\begin{array}{cc} 1, & \sqrt{u^{2}+v^{2}}\leq r_{c}\\ 0, & \mathrm{otherwise} \end{array}\right.,\;M_{\mathrm{HF}}=1-M_{\mathrm{LF}}$$

where $r_{c}$ denotes the cutoff radius determining the low–high frequency boundary, which is a learnable hyperparameter adjusted according to different HSI datasets.

The high- and low-frequency components in the frequency domain are reconstructed back to the spatial domain via inverse DFT to facilitate subsequent feature extraction and modeling:

(8) $$P_{\mathrm{LF}}=F^{-1}\left(F⊙M_{\mathrm{LF}}\right),\;P_{\mathrm{HF}}=F^{-1}\left(F⊙M_{\mathrm{HF}}\right)$$

where ⊙ is element-wise multiplication. $P_{\mathrm{LF}}$ and $P_{\mathrm{HF}}$, respectively, represent the low-frequency structural component and high-frequency detail component of the original patch.

To fully capture the distinct characteristics of different frequency components, each frequency component is fed into an independent Mamba network for long-range dependency modeling and feature enhancement:

(9) $$Z_{\mathrm{LF}}=f_{\mathrm{Mamba}-\mathrm{LF}}\left(P_{\mathrm{LF}}\right),\;Z_{\mathrm{HF}}=f_{\mathrm{Mamba}-\mathrm{HF}}\left(P_{\mathrm{HF}}\right)$$

where $Z_{\mathrm{LF}}\in R^{K\times K\times D}$ and $Z_{\mathrm{HF}}\in R^{K\times K\times D}$ are the low-frequency structural feature map and high-frequency detail feature map, respectively. D is the channel dimension after Mamba network.

The Mamba architecture, based on selective state-space modeling, efficiently captures long-range dependencies with linear complexity. Compared with Transformer-based models, Mamba reduces computational costs, allowing better feature representation for HSI classification.

### 3.4. Cross-Frequency Interaction Module

**Figure 3:**
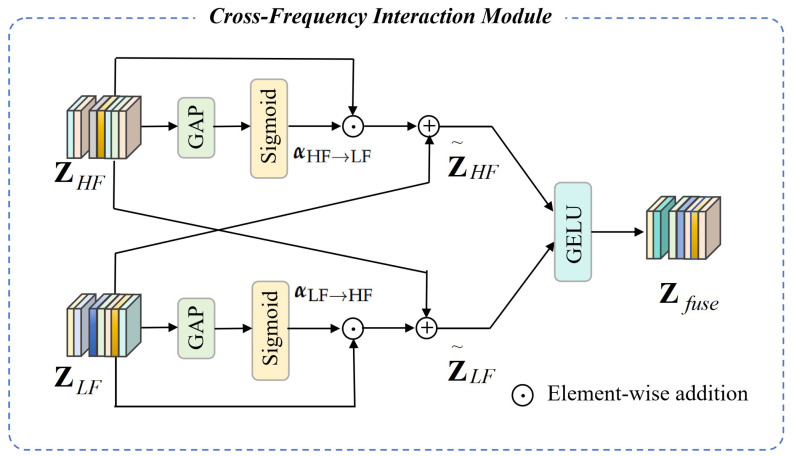
Architecture of the proposed cross-frequency interaction module. This module takes the enhanced low-frequency structural feature $Z_{\mathrm{LF}}$ and high-frequency detail feature $Z_{\mathrm{HF}}$ as inputs, first generates bidirectional dynamic attention weights, then implements adaptive bidirectional feature enhancement to realize mutual guidance between global structure and local details, and finally obtains the discriminative fused spectral–spatial feature $Z_{\mathrm{fuse}}$.

Although low-frequency and high-frequency branches capture complementary information, processing them independently may lead to insufficient representation. To address this, a cross-frequency interaction module is designed that enables bidirectional information exchange and adaptive feature fusion (Figure 3).

We first summarize global information from each branch using global average pooling (GAP) and project it through linear layers:

(10) $$\alpha_{\mathrm{LF}\to \mathrm{HF}}=\sigma \left(W_{1}\left[\mathrm{GAP}\left(Z_{\mathrm{LF}}\right)\right]\right),\;\alpha_{\mathrm{HF}\to \mathrm{LF}}=\sigma \left(W_{2}\left[\mathrm{GAP}\left(Z_{\mathrm{HF}}\right)\right]\right)$$

where σ(·) denotes the sigmoid activation function, $W_{1}\in R^{D\times D}$ and $W_{2}\in R^{D\times D}$ are learnable projection matrices, and GAP(·) represents the global average pooling operation. $\alpha_{\mathrm{LF}\to \mathrm{HF}}$ measures the contribution of low-frequency structural features to high-frequency detail feature enhancement, and $\alpha_{\mathrm{HF}\to \mathrm{LF}}$ measures the contribution of high-frequency texture features to low-frequency structural feature refinement.

Based on the generated dynamic attention weights, adaptive feature enhancement on each frequency branch is performed to achieve bidirectional information interaction:

(11) $${\tilde{Z}}_{\mathrm{HF}}=Z_{\mathrm{HF}}+\alpha_{\mathrm{LF}\to \mathrm{HF}}⊙Z_{\mathrm{LF}},\;{\tilde{Z}}_{\mathrm{LF}}=Z_{\mathrm{LF}}+\alpha_{\mathrm{HF}\to \mathrm{LF}}⊙Z_{\mathrm{HF}}$$

After bidirectional enhancement, the two frequency feature maps are concatenated and project them through a fusion layer to generate the final integrated spectral–spatial feature map:

(12) $$Z_{\mathrm{fuse}}=\phi \left([{\tilde{Z}}_{\mathrm{LF}}\;||\;{\tilde{Z}}_{\mathrm{HF}}]W_{f}\right)$$

where || denotes concatenation, ϕ(·) represents a GELU activation, and $W_{f}$ is a learnable fusion matrix. The GELU activation is mathematically defined as:

(13) $$\phi \left(x\right)=x\cdot \Phi \left(x\right)=0.5x\left(1+\mathrm{erf}\left(\frac{x}{\sqrt{2}}\right)\right)$$

where ϕ(x) is the cumulative distribution function of the standard normal distribution, and erf denotes the Gaussian error function. Unlike traditional piecewise activation functions (e.g., ReLU), GELU introduces soft stochasticity by weighting input values based on their probability of being active, which effectively alleviates overfitting and improves the generalization ability of deep learning models fo feature learning.

Finally, the integrated feature map is input into the classification head to obtain the final classification prediction. The classification head consists of a global average pooling layer, a fully connected layer, and a softmax activation function:

(14) $$y_{i}=\mathrm{Softmax}\left(W_{c}\cdot \mathrm{Pool}\left(Z_{\mathrm{fuse}}\right)+b_{c}\right)$$

where Pool(·) is the global average pooling operation and $W_{c}\in R^{D\times C}$ and $b_{c}\in R^{C}$ are the weight matrix and bias vector of the fully connected layer, respectively. To avoid noise addition during cross-frequency interaction, we adopt the dynamic attention weights generated by global average pooling which can adaptively weight the contribution of different frequency features, reducing the impact of noise. In addition, the GELU activation function in the fusion layer has a slight regularization effect, suppressing the propagation of noise components.

### 3.5. Frequency Alignment Loss

Although the cross-frequency interaction module promotes information exchange between high- and low-frequency features, during the training process, the two frequency branches may still converge to disjoint feature subspaces due to differences in the characteristics of the structural and texture features. To address this issue, a frequency alignment loss function is designed to explicitly constrain the distribution consistency and feature complementarity between high- and low-frequency features.

The total loss function of the HL-Mamba is a weighted combination of the cross-entropy loss ($L_{\mathrm{cls}}$) and the frequency alignment loss ($L_{\mathrm{align}}$). Among them, the cross-entropy loss is used to supervise the classification performance of model, ensuring that the integrated features can accurately distinguish different land-cover classes:

(15) $$L_{\mathrm{cls}}=-\frac{1}{N}\sum_{i=1}^{N}\sum_{c=1}^{C}y_{i,c}\log\left({\hat{y}}_{i,c}\right)$$

where *N* is the number of training samples and $y_{i,c}$ and ${\hat{y}}_{i,c}$ denote the ground truth label and predicted probability of the *i*-th sample for the *c*-th class, respectively.

The frequency alignment loss is designed to constrain the distribution consistency of high- and low-frequency features. We first normalize the high- and low-frequency feature maps to eliminate the impact of different feature scales, then calculate the L2 distance between the normalized features, and finally take the average of all samples as the alignment loss:

(16) $$L_{\mathrm{align}}=\frac{1}{N}\sum_{i=1}^{N}{∥\mathrm{Norm}\left(Z_{\mathrm{LF}}^{\left(i\right)}\right)-\mathrm{Norm}\left(Z_{\mathrm{HF}}^{\left(i\right)}\right)∥}_{2}^{2}$$

where Norm(·) denotes the normalization operation and $Z_{\mathrm{LF}}^{\left(i\right)}$ and $Z_{\mathrm{HF}}^{\left(i\right)}$ are the low-frequency and high-frequency feature maps of the *i*-th sample, respectively. Minimizing this loss can force the high- and low-frequency features to be distributed in similar subspaces, ensuring semantic consistency, while the differences in features are retained to maintain complementarity.

The total loss function of the network is defined as:

(17) $$L_{\mathrm{total}}=L_{\mathrm{cls}}+\lambda L_{\mathrm{align}}$$

where λ is a hyperparameter controlling the balance between the classification accuracy and the inter-frequency feature coherence. Through experiments, we set λ=0.2 to ensure that the frequency alignment loss can effectively promote feature consistency without compromising the classification performance.

## 4. Results

### 4.1. Experimental Setup

**Figure 4:**
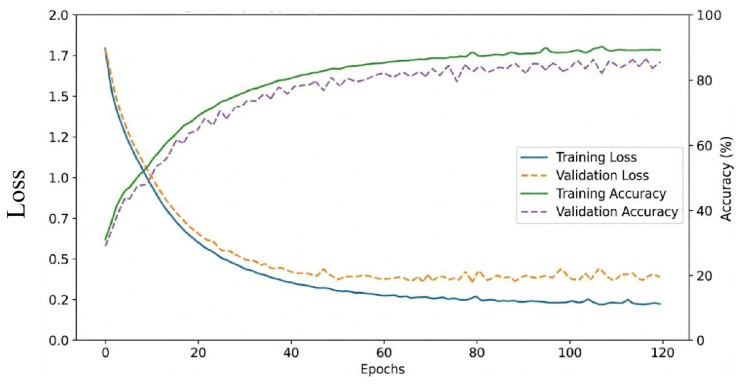
Training and evaluation curves on the Indian Pines dataset, which include training loss, validation loss, training accuracy, and test accuracy, intuitively reflecting the convergence process, fitting status, and generalization performance of the proposed HL-Mamba.

All experiments are implemented using PyTorch 2.2 and are conducted on a NVIDIA RTX 4090 GPU. The Adam optimizer is used for model training with an initial learning rate of $1\times 10^{-3}$ and a batch size of 64. During data preprocessing, PCA is used to reduce the spectral dimension to 30 to preserve key spectral information while reducing redundancy. The network depth is configured as three Mamba modules, with a total of six Mamba modules for feature extraction in the dual branches. The loss weight λ in the total loss function is adjusted through experiments, and the value was set to 0.2. The training curves of the Indian Pines dataset are shown in Figure 4. All models are trained for a maximum of 100 epochs, and the model with the highest validation accuracy is selected for testing.

### 4.2. Evaluation Metrics

Three metrics are used to quantitatively evaluate the classification performance, i.e., overall accuracy (OA), average accuracy (AA), and Kappa coefficient. OA measures the global classification performance as the ratio of correctly classified pixels to the total number of pixels, which is mathematically defined as:

(18) $$\mathrm{OA}=\frac{\sum_{i=1}^{C}n_{ii}}{\sum_{i=1}^{C}\sum_{j=1}^{C}n_{ij}}\times 100\%$$

where *C* denotes the total number of land cover categories, $n_{ii}$ represents the number of pixels correctly classified into class *i*, and $n_{ij}$ is the number of pixels belonging to class *i* but predicted as class *j*. AA addresses category imbalance by calculating the arithmetic mean of class-wise accuracies, reflecting the model’s ability to classify each category evenly. Its formulation is given by:

(19) $$\mathrm{AA}=\frac{1}{C}\sum_{i=1}^{C}\left(\frac{n_{ii}}{\sum_{j=1}^{C}n_{ij}}\right)\times 100\%$$

The Kappa coefficient is a robust metric that evaluates the agreement between predicted results and ground truth while eliminating the impact of random classification, providing a more reliable assessment beyond mere chance. It is calculated as:

(20) $$\mathrm{Kappa}=\frac{\mathrm{OA}-P_{e}}{1-P_{e}}$$

where $P_{e}=\frac{1}{N^{2}}\sum_{i=1}^{C}\left(\sum_{j=1}^{C}n_{ij}\right)\left(\sum_{j=1}^{C}n_{ji}\right)$ is the expected accuracy due to random chance, and $N=\sum_{i=1}^{C}\sum_{j=1}^{C}n_{ij}$ is the total number of test pixels.

### 4.3. Performance Comparison

**Figure 5:**
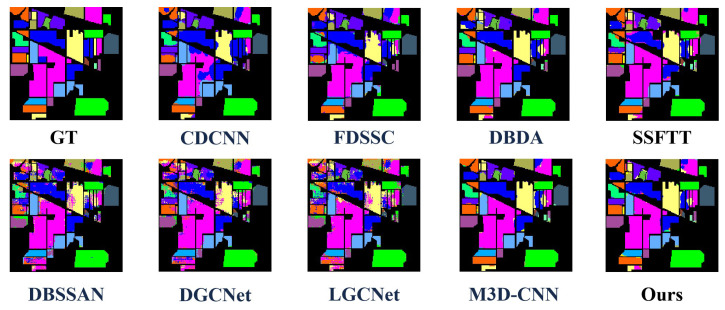
Classification maps on the Indian Pines dataset, containing results of CDCNN, FDSSC, DBDA, SSFTT, DBSSAN, DGCNet, LGCNet, M3DCNN, and the proposed method, showing the better classification performance of the proposed method.

**Figure 6:**
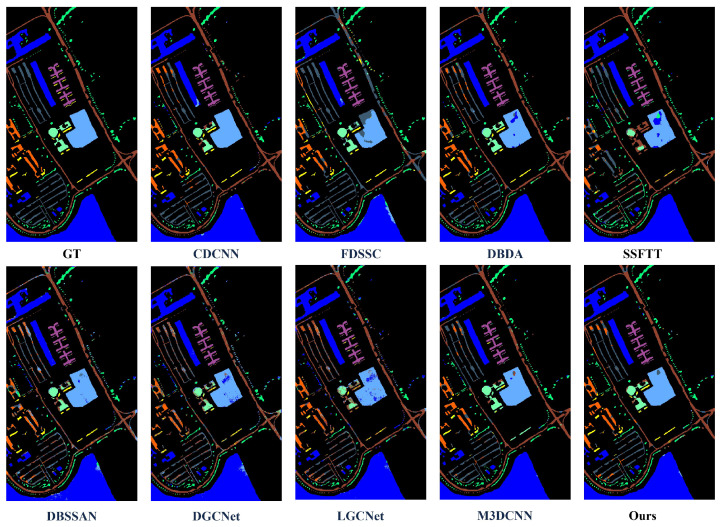
Classification maps on the Pavia University dataset, containing results of CDCNN, FDSSC, DBDA, SSFTT, DBSSAN, DGCNet, LGCNet, M3DCNN, and the proposed method, showing the better classification performance of the proposed method.

**Figure 7:**
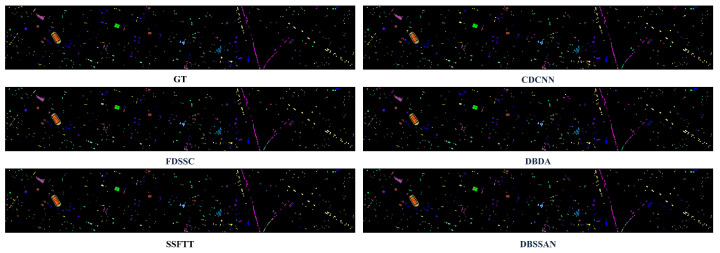

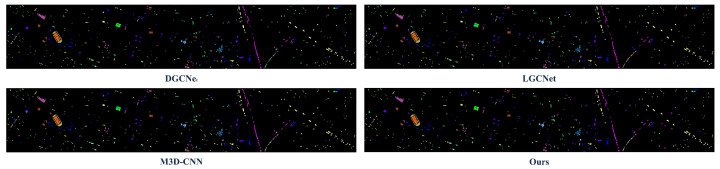
Classification maps on the Houston dataset, containing results of CDCNN, FDSSC, DBDA, SSFTT, DBSSAN, DGCNet, LGCNet, M3DCNN, and the proposed method, showing the better classification performance of the proposed method.

**Figure 8:**
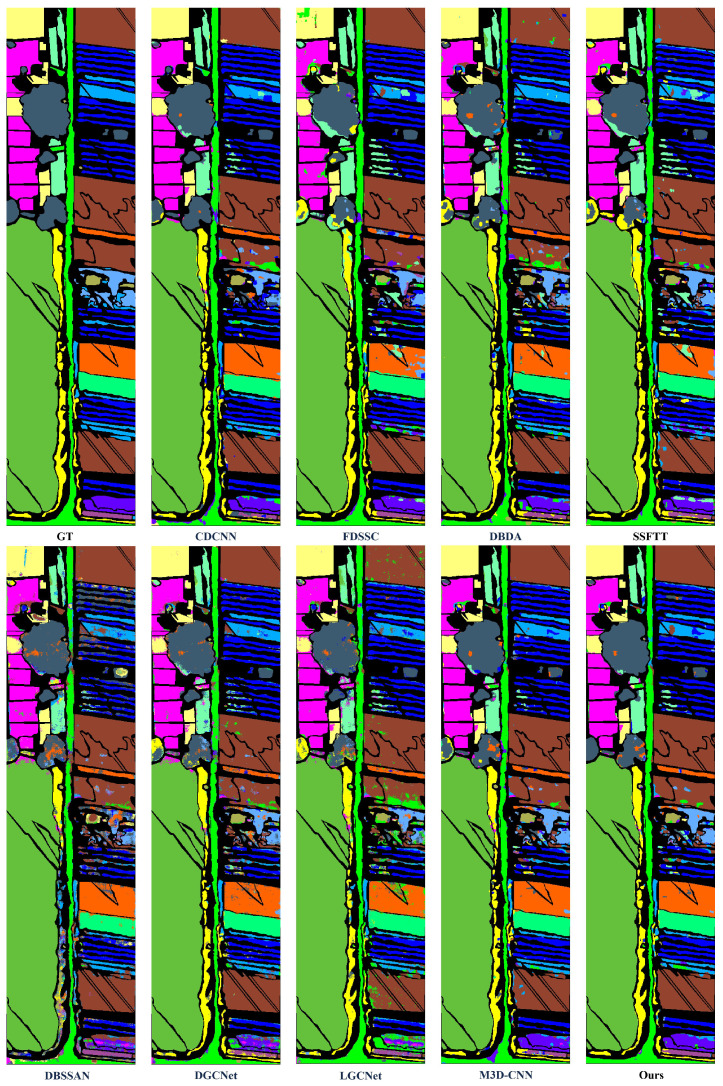
Classification maps on the WHU-Hi-HanChuan dataset, containing results of CDCNN, FDSSC, DBDA, SSFTT, DBSSAN, DGCNet, LGCNet, M3DCNN, and the proposed method, showing the better classification performance of the proposed method.

To further validate the performance of HL-Mamba, we compare it with eight HSI classification methods, including CNN-based methods (CDCNN [42], FDSSC [43], M3D-CNN [44], DGCNet [45], LGCNet [46]) and spectral–spatial feature fusion methods (DBDA [47], DBSSAN [48], SSFTT [22]). The quantitative results on the four datasets are summarized in Table 2, Table 3, Table 4 and Table 5, and the qualitative classification maps are shown in Figure 5, Figure 6, Figure 7 and Figure 8. The optimal values for OA, AA, and Kappa are indicated in bold. The second-best values are indicated with double underlines, and the third-best values are indicated with a single underline.

#### 4.3.1. Quantitative Results and Analysis

1. Indian Pines Dataset: As shown in Table 2, HL-Mamba achieves the highest OA (94.07%), AA (90.40%), and Kappa (93.25%) among all compared methods. Compared with the second-best method DBDA (OA = 93.44%), HL-Mamba improves OA by 0.63%. The key reason is that the high–low frequency decomposition effectively separates the global structure and edge details of crops, and the cross-frequency interaction module enhances the discriminability of spectrally similar classes (e.g., Alfalfa and Oats), which is difficult for comparison methods to achieve. Notably, HL-Mamba shows significant advantages in class-level accuracy for challenging classes with high spectral similarity. For example, the accuracy of Alfalfa (a minority class) reaches 53.78%, which is 2.45% higher than DBDA (51.33%) and far superior to CDCNN (0.89%) and FDSSC (24.00%). For Oats, HL-Mamba achieves 79.47% accuracy, outperforming M3DCNN (78.42%) and DBDA (73.16%). This indicates that the frequency-aware feature learning of HL-Mamba effectively enhances the discrimination of spectrally similar classes.
2. Pavia University Dataset: Table 3 presents the quantitative results of HL-Mamba and comparison methods on the Pavia University dataset. This dataset is characterized by high resolution and large samples for most classes, which demands models to effectively capture fine-grained spatial details in processing large-scale data. HL-Mamba achieves the highest AA of 90.66% and Kappa of 91.79%. In terms of class-level accuracy, HL-Mamba shows remarkable performance in classes with similar spatial textures and spectral properties. For example, the Asphalt and Bitumen classes, which are easily misclassified due to their similar gray-scale textures in spatial images, HL-Mamba achieves accuracies of 90.82% and 95.18%, respectively. For the Shadows class, a typical low-frequency structural class with large intra-class variability, HL-Mamba achieves an accuracy of 82.72%. This advantage comes from the low-frequency branch’s efficient modeling of global structural context, which accurately identifies shadow regions. For the high-resolution urban scene of Pavia University, the key advantage lies in the HLFDMM’s ability to capture fine-grained spatial details (via high-frequency branch) and global structural consistency (via low-frequency branch). For example, the Asphalt class (easily confused with Bitumen due to similar gray textures) achieves 90.82% accuracy, and the Shadows class (with large intra-class variability) reaches 82.72% accuracy, far higher than M3DCNN’s 63.55%. This is because the low-frequency branch models the global context of shadow regions, while the cross-frequency interaction module refines edge details of urban structures, effectively mitigating misclassification caused by spatial similarity.
3. Houston Dataset: The Houston dataset is a challenging urban hyperspectral dataset with complex mixed pixels, shadows, and occlusions, requiring models to extract spectral–spatial feature better. As shown in Table 4, HL-Mamba achieves the highest OA of 87.32%, AA of 88.18%, and Kappa of 86.29%. The dataset’s challenges (mixed pixels, shadows, occlusions) are addressed by the synergy of CFIM and FAL: for the Commercial class (affected by high-rise building shadows), HL-Mamba’s accuracy (63.68%) is 3.45% higher than DBDA (60.23%); for the Parking Lot 2 class (small-scale and easily mixed with roads), accuracy reaches 86.90%, outperforming the second-best FDSSC (80.80%). The FAL enhances feature invariance to illumination variations, while the CFIM fuses structural (low-frequency) and texture (high-frequency) features, making the model robust to urban interference factors. For classes affected by shadows and occlusions, such as Commercial and Railway, HL-Mamba shows significant advantages. The Commercial class, which is often misclassified due to the shadow of high-rise buildings, achieves an accuracy of 63.38% with HL-Mamba, 3.45% higher than DBDA (92.48%). This is because the frequency alignment loss enhances the invariance of features to illumination variations, making the model less sensitive to shadow-induced spectral distortions.
4. WHU-Hi-HanChuan Dataset: As a large-scale agricultural and suburban hyperspectral dataset, WHU-Hi-HanChuan features a vast scene size and high spectral diversity, testing the model’s performance. Table 5 presents the quantitative results, where HL-Mamba achieves the highest OA of 95.28%, AA of 89.81%, and Kappa of 94.47%, outperforming the second-ranked M3DCNN (OA = 94.26%, Kappa = 993.28%). For crop classes with overlapping spectral bands (e.g., Soybean and Sorghum), HL-Mamba achieves 92.70% and 97.42% accuracy, respectively, benefiting from HLFDMM’s effective decomposition of spectral–spatial features. For the Water-spinach class (a minority crop with sparse samples), accuracy reaches 85.78%, 15.31% higher than DBDA (70.75%), as the cross-frequency interaction module aggregates discriminative details from high-frequency features, compensating for the lack of training samples. For agricultural crop classes with similar spectral characteristics, such as Soybean and Sorghum, HL-Mamba achieves accuracies of 97.42% and 85.78%, respectively. This demonstrates that the frequency-aware feature learning effectively extracts discriminative spectral features from overlapping spectral bands of different crops.

#### 4.3.2. Visualization Results and Analysis

Figure 5, Figure 6, Figure 7 and Figure 8 illustrate classification maps generated by the proposed method and baselines. Visually, the proposed method yields smoother boundaries and fewer misclassified pixels, especially along class edges and shadowed regions. The high-frequency branch captures subtle texture transitions, while the low-frequency branch preserves large-area semantic consistency. The resulting maps exhibit clear object details.

1. Indian Pines Dataset: Qualitatively, Figure 5 visualizes the classification maps of HL-Mamba and comparison methods on the Indian Pines dataset, which is dominated by agricultural land cover with similar crop types. The classification map of the HL-Mamba exhibits sharp edge details across the entire scene. In contrast, CDCNN and FDSSC suffer from severe salt-and-pepper noise in minority class regions, and their classification results show blurred boundaries between Oats and surrounding grassland classes. For the Grass-pasture-mowed class, a sparse category prone to misclassification, HL-Mamba clearly demarcates its distribution with minimal pixel confusion. This visual superiority stems from the CFIM and frequency-aware feature learning of HL-Mamba. The high-frequency branch captures fine texture differences between similar crops, while the low-frequency branch anchors the global structural distribution of sparse classes.
2. Pavia University: Qualitatively, the classification map of HL-Mamba (Figure 6) shows clear edge details and minimal misclassification noise compared to other methods. For instance, in the buildings, HL-Mamba accurately distinguishes the classes with no obvious mixed pixels, while CDCNN and FDSSC exhibit slight misclassification in these edge regions. Additionally, HL-Mamba effectively suppresses the “salt-and-pepper” noise in the classification map of the Gravel class, which is attributed to the CFIM integration of global and local features, enhancing the spatial consistency of classification results.
3. Houston Dataset: Figure 7 presents the qualitative classification results on the Houston dataset, a complex urban scene with mixed pixels. The classification map of HL-Mamba accurately delineates the boundaries of distinct urban functional areas, with no obvious mixed pixels in the transition zones. For shadow-affected regions, HL-Mamba maintains consistent classification accuracy without spectral distortion-induced misclassification, while DBDA and M3DCNN show scattered misclassified pixels in these shadowed areas. Notably, HL-Mamba precisely identifies small-scale high-texture objects embedded in complex urban landscapes, with clear and complete details of their shapes. Additionally, for the Healthy grass and Stressed grass classes with high intra-class variability, HL-Mamba’s classification map shows smooth spatial transitions between the two grass types, reflecting the effective fusion of global structure and local details via the CFIM.
4. WHU-Hi-HanChuan Dataset: Qualitatively, Figure 8 shows that HL-Mamba’s classification map accurately captures the large-scale distribution of agricultural crops and urban areas, with no obvious misclassification in the transition zones between Strawberry fields and Soybean fields. Compared to CDCNN and FDSSC, HL-Mamba’s classification map has better spatial continuity, especially in the Water class, where the boundaries of water bodies are clearly delineated without being affected by surrounding vegetation shadows. This confirms that HL-Mamba is well-suited for large-scale HSI classification tasks.

#### 4.3.3. Computational Complexity

**Figure 9:**
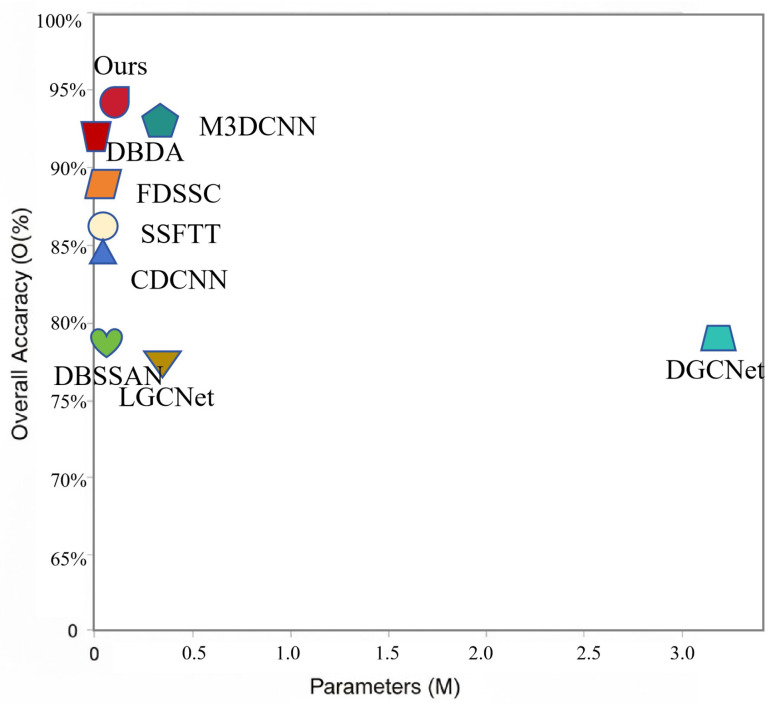
The visualization results of the number of parameters and the of OA for each method in the Indian Pines dataset, containing CDCNN, FDSSC, DBDA, SSFTT, DBSSAN, DGCNet, LGCNet, M3DCNN, and the proposed method.

To evaluate the efficiency of HL-Mamba, we analyzed its model parameters, computational complexity (FLOPs), and test time on the Indian Pines dataset and compared them with eight methods under the same hardware platform. As shown in Table 6, HL-Mamba achieves an excellent balance between classification performance and model efficiency. The visualization results of the number of parameters and the of OA for each method in the Indian Pines dataset are shown in Figure 9. It has a compact parameter count of 0.2957 M, a low FLOP count of 0.0354 G, and a fast average test time of 0.2934 s, which demonstrates its lightweight and high-efficiency characteristics for HSI classification. Compared with other methods, HL-Mamba shows obvious advantages in reducing computational overhead and accelerating inference. HL-Mamba significantly reduces both model parameters and inference latency, which benefits from the linear-complexity Mamba backbone, frequency decomposition-based redundant computation reduction, and a lightweight cross-frequency interaction mechanism. Although slightly higher in parameters and FLOPs than some extremely lightweight methods (e.g., DBDA with 0.0389 M parameters, M3DCNN with 0.0095 G FLOPs), HL-Mamba outperforms these methods by a large margin in classification accuracy (OA) on the Indian Pines dataset, which verifies its trade-off of performance and efficiency.

## 5. Discussion

To validate the effectiveness of key components in HL-Mamba and the influence of critical hyperparameters, we carried out ablation experiments on the Indian Pines dataset. The Indian Pines dataset was selected due to its high class diversity and spectral similarity challenges, which can effectively highlight the performance of model. Two sets of ablation experiments were designed. The first was a sensitivity analysis of hyperparameter λ and the number of Mamba modules. The second was module ablation. These experiments aimed to validate the contributions of the high–low frequency decomposition Mamba module (HLFDMM), CFIM, and frequency alignment loss (FAL).

### 5.1. Sensitivity Analysis of Hyperparameter

To investigate the sensitivity of the model’s classification performance to the number of Mamba modules, experiments were conducted with the number of Mamba modules set to 2, 3, 4, and 5, respectively. The results are presented in Table 7. It can be observed that the classification metrics exhibit a trend of first increasing and then decreasing with the increase in the number of Mamba modules. When the number of Mamba modules is 3, the model achieves the optimal performance, with OA reaching 94.07%, AA 90.40%, and Kappa 93.25%. This indicates that an appropriate increase in the number of Mamba modules can enhance the model’s ability, thereby improving HSI classification accuracy. However, when the number of modules exceeds 3, all metrics show a decline. This phenomenon may be attributed to the excessive complexity of the model caused by redundant Mamba modules, leading to overfitting and a reduction in feature representation efficiency. Therefore, the optimal number of Mamba modules for the proposed model is determined to be 3.

The loss weight λ balances the classification loss $L_{\mathrm{cls}}$ and the frequency alignment loss $L_{\mathrm{align}}$. To determine the optimal λ, experiments are conducted with λ varying from 0 to 0.6 at intervals of 0.1. The results are summarized in Table 8. When λ=0 (i.e., no frequency alignment loss), the OA is 92.86%, AA is 88.57%, and Kappa is 91.85%. With the increase of λ from 0 to 0.2, all metrics gradually improve, reaching the highest performance (OA = 94.07%, AA = 90.40%, Kappa = 93.25%) at λ=0.2. This indicates that the frequency alignment loss effectively enhances the consistency and complementarity between high- and low-frequency features, thereby improving HSI classification performance. However, when λ exceeds 0.2, the metrics begin to decrease. For example, when λ=0.6, OA drops to 92.53%, which is lower than the result at λ=0.2. This is because a large λ over-constrains the feature distribution of the two frequency branches, leading to the loss of complementary details between high- and low-frequency features. Thus, setting λ=0.2 achieves the optimal balance between classification accuracy and inter-frequency feature.

### 5.2. Module Ablation

To validate the contributions of the high–low frequency decomposition Mamba module (HLFDMM), CFIM, and frequency alignment loss (FAL), module ablation experiments were conducted, as shown in Table 9. The baseline model without any of the three modules achieves an OA of 89.25%, AA of 84.11%, and Kappa of 87.98%. Specifically, enabling only HLFDMM yields the most significant standalone performance improvement (OA = 92.86%), confirming its core role in decomposing HSIs into discriminative high–low frequency features to capture global structures and fine-grained details. In contrast, enabling only CFIM improves OA to 90.18% by facilitating cross-frequency feature interaction, while enabling only FAL raises OA to 89.76% by aligning feature distributions across frequency branches; both exhibit limited standalone gains because CFIM merely establishes interaction without relying on effective frequency decomposition and FAL lacks the support of decoupled high–low frequency features. Furthermore, among dual-module combinations, the HLFDMM + CFIM configuration performs best (OA = 93.69%), as CFIM can fully exploit the discriminative value of HLFDMM-decomposed features to enhance inter-frequency complementarity, while the HLFDMM + FAL combination also delivers solid results (OA = 93.24%) by integrating decomposition and alignment. Notably, activating all three modules achieves the optimal performance (OA = 94.07%, AA = 90.40%, Kappa = 93.25%), demonstrating that only when combined with HLFDMM, whose frequency decomposition lays the foundation for discriminative feature learning, can CFIM fully leverage the complementary advantages of different frequency features and FAL effectively enhance the consistency of decoupled features, ultimately achieving synergistic performance improvement in HSI classification.

### 5.3. Limitations

Despite the promising performance of the proposed HL-Mamba model demonstrated by the above experiments, it still has limitations. The frequency decomposition strategy adopted in HLFDMM is fixed, and it lacks adaptability to different types of ground objects in HSIs, which may restrict the model’s ability to capture task-specific frequency features.

## 6. Conclusions

This study makes three key contributions that integrate frequency-domain decomposition and Mamba to effectively address feature entanglement and computational redundancy, designing a CFIM to achieve bidirectional information complementation between high- and low-frequency features, and designing an FAL to enhance feature consistency. Specifically, by decomposing HSI into complementary low-frequency structural components and high-frequency edge detail components, and employing parallel Mamba branches to model different frequency component, the proposed method mitigates the problems of feature entanglement and information redundancy. Furthermore, through the bidirectional information flow between different frequency features established by the cross-frequency interaction module, and the consistency enhancement of frequency-domain features by the frequency alignment loss, HL-Mamba is able to learn more discriminative spectral–spatial representations and significantly enhance the HSI classification capability. Extensive experiments conducted on four public benchmark datasets demonstrate that the proposed HL-Mamba consistently achieves state-of-the-art classification performance while maintaining high computational efficiency, benefiting from the linear-complexity Mamba backbone and lightweight cross-frequency interaction module. Ablation studies and visualization analyses further confirm the effectiveness of each core component of the overall framework. Future research will focus on developing adaptive frequency separation schemes, exploring multi-scale frequency fusion, and extending the model to large-scale or real-time remote sensing applications.

## Figures and Tables

**Table 1:** Detailed category information of four datasets.

**Indian Pines Dataset**				**Houston Dataset**			
**Class**	**Class Name**	**Train**	**Test**	**Class**	**Class Name**	**Train**	**Test**
1	Alfalfa	1	45	1	Healthy grass	13	1238
2	Corn-notill	43	1385	2	Stressed grass	13	1241
3	Corn-mintill	25	805	3	Synthetic grass	7	690
4	Corn	7	230	4	Trees	12	1232
5	Grass-pasture	14	469	5	Soil	12	1230
6	Grass-trees	22	708	6	Water	3	322
7	Grass-pasture-mowed	1	27	7	Residential	13	1255
8	Hay-windrowed	14	464	8	Commercial	12	1232
9	Oats	1	19	9	Road	13	1239
10	Soybean-notill	29	943	10	Highway	12	1215
11	Soybean-mintill	73	2382	11	Railway	12	1223
12	Soybean-clean	18	575	12	Parking Lot 1	12	1221
13	Wheat	6	199	13	Parking Lot 2	5	464
14	Woods	38	1227	14	Tennis Court	4	424
15	Buildings-Grass-Trees-Drives	12	374	15	Running Track	7	653
16	Stone-Steel-Towers	3	90	-	Total	150	14,879
-	Total	307	9942				
**Pavia University Dataset**				**WHU-Hi-HanChuan Dataset**			
**Class**	**Class Name**	**Train**	**Test**	**Class**	**Class Name**	**Train**	**Test**
1	Asphalt	33	6598	1	Strawberry	224	44,511
2	Meadows	93	18,556	2	Cowpea	114	
3	Gravel	10	2089	3	Soybean	51	10,236
4	Trees	15	3049	4	Sorghum	27	5326
5	Painted metal sheets	7	1338	5	Water-spinach	6	1194
6	Bare Soil	25	5004	6	Watermelon	23	4510
7	Bitumen	7	1323	7	Greens	29	5874
8	Self-Blocking Bricks	18	3664	8	Trees	90	17,888
9	Shadows	5	942	9	Grass	47	9422
-	Total	213	42,563	10	Red-roof	52	10,464
				11	Gray-roof	84	16,827
				12	Plastic	18	3661
				13	Bare-soil	46	9070
				14	Road	93	18,467
				15	Bright-object	6	1130
				16	Water	377	75,024
				-	Total	1287	256,243

**Table 2:** Classification results on the Indian Pines dataset.

Class	CDCNN	FDSSC	DBDA	SSFTT	DBSSAN	DGCNet	LGCNet	M3DCNN	Ours
Alfalfa	0.89	24.00	51.33	38.67	15.56	20.00	20.67	42.22	**53.78**
Corn-notill	78.06	86.82	92.35	81.86	73.60	73.69	72.58	92.09	**92.53**
Corn-mintill	88.05	84.17	91.13	86.41	61.13	66.45	63.65	90.93	**92.37**
Corn	79.70	80.52	87.78	77.26	57.74	52.83	55.35	89.87	**90.70**
Grass-pasture	**90.30**	87.74	91.62	83.54	67.01	74.03	72.81	87.80	90.00
Grass-trees	91.27	91.81	**98.81**	94.04	94.65	96.43	94.83	97.16	96.84
Grass-pasture-mowed	12.59	56.67	90.37	50.37	20.74	27.41	18.52	90.74	**93.33**
Hay-windrowed	97.41	97.33	99.20	97.76	98.43	98.36	97.56	98.97	**99.46**
Oats	0.00	32.11	73.16	67.37	11.58	25.26	17.37	78.42	**79.47**
Soybean-notill	79.95	87.25	**90.36**	80.84	69.09	71.63	67.30	89.85	89.70
Soybean-mintill	81.38	91.05	95.27	90.34	82.67	83.72	83.74	95.26	**95.93**
Soybean-clean	84.23	79.97	82.12	78.00	51.10	54.35	52.05	87.01	**90.38**
Wheat	89.65	95.08	**98.14**	89.40	90.90	94.07	89.50	97.74	98.04
Woods	**98.21**	97.39	98.32	92.67	97.07	97.46	95.83	96.50	98.04
Buildings-Grass-Trees-Drives	84.17	90.11	91.39	88.56	75.91	74.49	69.73	92.78	**94.92**
Stone-Steel-Towers	78.00	87.33	**97.00**	84.00	84.78	82.67	80.67	87.44	90.89
OA (%)	84.93	89.13	$\underline{\underline{93.44}}$	86.97	77.92	79.49	77.89	93.06	**94.07**
AA (%)	70.87	79.33	$\underline{\underline{89.27}}$	80.07	65.75	68.30	65.76	88.42	**90.40**
Kappa (%)	83.00	87.62	$\underline{\underline{92.52}}$	85.15	74.73	76.52	74.64	92.11	**93.25**

**Table 3:** Classification results on the Pavia University dataset.

Class	CDCNN	FDSSC	DBDA	SSFTT	DBSSAN	DGCNet	LGCNet	M3DCNN	Ours
Asphalt	**96.30**	88.17	87.45	90.09	81.52	84.69	85.85	89.85	90.82
Meadows	95.36	99.15	**99.52**	99.26	98.72	99.29	89.30	99.41	99.28
Gravel	73.62	**77.45**	82.16	69.51	48.86	64.13	57.58	77.14	76.09
Trees	80.65	84.45	**89.99**	86.76	70.43	74.36	70.37	84.68	85.89
Painted metal sheets	99.57	99.32	99.78	98.83	99.19	99.51	99.13	99.78	**99.79**
Bare Soil	90.76	96.47	94.01	96.38	86.92	85.19	85.13	97.09	**97.78**
Bitumen	86.02	**96.53**	97.99	82.00	59.06	65.31	55.59	92.80	95.18
Self-Blocking Bricks	81.99	**90.44**	81.56	78.00	64.64	65.28	60.70	85.56	83.02
Shadows	77.31	**83.30**	80.61	72.52	59.84	71.60	64.17	63.55	82.70
OA (%)	91.13	**93.84**	93.46	92.17	85.18	87.27	81.61	93.33	$\underline{\underline{93.82}}$
AA (%)	86.84	$\underline{\underline{90.59}}$	90.34	85.93	74.35	78.82	74.20	87.76	**90.66**
Kappa (%)	88.57	91.06	$\underline{\underline{91.32}}$	89.59	79.94	82.80	76.53	91.14	**91.79**

**Table 4:** Classification results on the Houston dataset.

Class	CDCNN	FDSSC	DBDA	SSFTT	DBSSAN	DGCNet	LGCNet	M3DCNN	Ours
Healthy grass	81.39	88.34	90.35	86.95	86.79	89.99	89.39	**90.74**	90.55
Stressed grass	87.51	88.76	92.39	89.19	86.45	90.61	89.33	89.46	**93.22**
Synthetic grass	98.55	98.32	98.13	98.86	96.70	95.13	96.09	98.33	**98.94**
Trees	89.33	88.98	**91.78**	85.98	83.68	83.12	86.57	89.16	90.32
Soil	98.07	97.32	99.59	95.83	97.20	98.67	97.79	99.11	**99.62**
Water	79.19	78.76	**82.14**	75.90	66.96	76.34	72.42	71.93	81.34
Residential	**92.68**	86.69	84.32	85.22	68.13	75.78	78.21	80.96	84.72
Commercial	61.00	65.86	60.23	60.62	62.49	**67.82**	67.66	62.83	63.68
Road	**81.82**	79.45	74.61	74.47	65.85	63.54	69.23	73.61	79.01
Highway	89.53	90.25	90.62	**92.83**	84.05	85.45	86.53	91.65	85.99
Railway	**95.08**	93.07	92.31	91.10	81.29	85.41	83.70	89.90	89.22
Parking Lot 1	74.83	76.52	81.88	82.24	74.28	80.48	80.09	**83.76**	82.83
Parking Lot 2	73.47	80.80	86.51	80.28	81.08	82.50	82.56	86.55	**86.90**
Tennis Court	99.60	99.72	**100.00**	95.64	86.96	94.76	93.92	99.03	99.34
Running Track	**99.86**	99.40	97.64	97.92	97.00	99.25	99.65	99.51	96.98
OA (%)	86.32	86.83	$\underline{\underline{87.24}}$	85.70	80.64	83.68	84.27	86.50	**87.32**
AA (%)	86.79	87.48	$\underline{\underline{88.17}}$	86.20	81.26	84.59	84.88	87.10	**88.18**
Kappa (%)	85.21	85.77	$\underline{\underline{86.20}}$	84.54	79.06	82.36	82.99	85.41	**86.29**

**Table 5:** Classification results on the WHU-Hi-HanChuan dataset.

Class	CDCNN	FDSSC	DBDA	SSFTT	DBSSAN	DGCNet	LGCNet	M3DCNN	Ours
Strawberry	87.19	98.18	97.20	**98.58**	97.42	97.20	97.02	97.83	98.40
Cowpea	86.28	**94.65**	92.00	92.77	86.06	89.61	90.88	93.12	94.01
Soybean	82.19	88.24	88.92	**93.09**	88.32	88.60	88.33	89.92	92.70
Sorghum	94.44	96.73	97.05	95.84	95.31	97.10	96.29	96.03	**97.42**
Water-spinach	58.73	**86.38**	70.75	78.02	50.64	63.20	63.89	70.47	85.78
Watermelon	60.78	74.44	69.20	74.67	57.71	61.28	52.09	71.65	**76.06**
Greens	86.60	92.75	90.67	**93.02**	82.81	89.15	85.92	91.66	92.61
Trees	86.66	89.95	87.95	87.28	83.28	83.44	83.10	88.49	**90.79**
Grass	83.39	87.71	87.51	86.69	71.61	85.72	77.82	89.01	**90.12**
Red-roof	96.32	96.11	95.78	96.25	93.75	93.51	93.65	97.40	**97.80**
Gray-roof	93.44	96.51	95.82	97.17	94.23	95.28	93.39	96.26	**97.30**
Plastic	79.53	86.58	82.66	85.14	46.78	55.83	48.07	82.86	**88.01**
Bare-soil	72.14	73.45	76.86	73.82	68.80	70.72	66.62	**77.83**	77.76
Road	92.80	93.68	90.05	89.61	86.11	89.93	88.77	91.86	**93.73**
Bright-object	**68.92**	68.27	66.49	57.42	40.73	62.29	53.69	60.06	64.64
Water	99.04	99.56	99.63	**99.76**	99.44	99.68	99.66	99.68	99.75
OA (%)	90.21	$\underline{\underline{94.60}}$	93.62	94.18	90.25	92.07	91.07	94.26	**95.28**
AA (%)	83.03	$\underline{\underline{88.95}}$	86.78	87.44	77.69	82.66	79.95	87.13	**89.81**
Kappa (%)	88.63	$\underline{\underline{93.67}}$	92.53	93.18	88.56	90.70	89.53	93.28	**94.47**

**Table 6:** Model parameters and computational complexity comparison.

Metric	CDCNN	FDSSC	DBDA	SSFTT	DBSSAN	DGCNet	LGCNet	M3DCNN	Ours
Parameters (M)	0.3022	0.1839	0.0389	0.1485	0.0721	3.3664	0.3782	0.4412	0.2957
FLOPs (G)	0.0506	0.4964	0.0556	0.0114	0.1398	0.0344	0.0193	0.0095	0.0354
Test Time (s)	0.3979	2.0812	0.9961	0.5796	1.4631	23.5910	29.6271	4.2803	0.2934

**Table 7:** Classification performance under different numbers of Mamba modules on the Indian Pines dataset.

Number of Mamba Modules	OA (%)	AA (%)	Kappa (%)
2	92.15	87.32	90.98
3 (Ours)	**94.07**	**90.40**	**93.25**
4	93.58	89.76	92.71
5	92.89	88.61	91.88

**Table 8:** Classification performance under different loss weight λ on the Indian Pines dataset.

λ	OA (%)	AA (%)	Kappa (%)
0.0	92.86	88.57	91.85
0.1	93.52	89.63	92.58
0.2 (Ours)	**94.07**	**90.40**	**93.25**
0.3	93.84	89.95	92.99
0.4	93.21	89.12	92.27
0.5	92.93	88.76	91.93
0.6	92.53	88.24	91.48

**Table 9:** Classification results of different module combinations on the Indian Pines dataset.

HLFDMM	CFIM	FAL	OA (%)	AA (%)	Kappa (%)
×	×	×	89.25	84.11	87.98
✓	×	×	92.86	88.57	91.85
×	✓	×	90.18	85.62	89.03
×	×	✓	89.76	84.83	88.59
✓	✓	×	93.69	89.92	92.88
✓	×	✓	93.24	89.31	92.37
×	✓	✓	90.87	86.45	89.81
✓	✓	✓	**94.07**	**90.40**	**93.25**

## Data Availability

Data and code are publicly available at https://github.com/tengye663/HL-Mamba, accessed on 25 February 2026.

## Abbreviations

The following abbreviations are used in this manuscript:

HSI	Hyperspectral image
CNN	Convolutional neural network
PCA	Principal component analysis
RNN	Recurrent neural network
HL-Mamba	High–low frequency interaction Mamba network
DFT	Discrete Fourier transform
